# CBIR-SAR System Using Stochastic Distance

**DOI:** 10.3390/s23136080

**Published:** 2023-07-01

**Authors:** Alcilene Dalília Sousa, Pedro Henrique dos Santos Silva, Romuere Rodrigues Veloso Silva, Francisco Alixandre Àvila Rodrigues, Fatima Nelsizeuma Sombra Medeiros

**Affiliations:** 1 Informatics Systems, Federal University of Piaui, Picos 64607-825, Piaui, Brazil; romuere@ufpi.edu.br; 2 Teleinformatics Engineering, Federal University of Ceara, Fortaleza 60455-970, Ceara, Brazil; fsombraufc@gmail.com; 3 Computer Engineering, Federal University of Ceara, Fortaleza 60455-970, Ceara, Brazil; pedrosantossilva05@alu.ufc.br; 4 Computational Mathematics, Federal University of Cariri, Juazeiro do Norte 63048-080, Ceara, Brazil; alixandre.avila@ufca.edu.br

**Keywords:** SAR, CBIR, stochastic distance, fast log-cumulants method, maximum likelihood estimation

## Abstract

This article proposes a system for Content-Based Image Retrieval (CBIR) using stochastic distance for Synthetic-Aperture Radar (SAR) images. The methodology consists of three essential steps for image retrieval. First, it estimates the roughness ($\hat{\alpha }$) and scale ($\hat{\gamma }$) parameters of the $G_{I}^{0}$ distribution that models SAR data in intensity. The parameters of the model were estimated using the Maximum Likelihood Estimation and the fast approach of the Log-Cumulants method. Second, using the triangular distance, CBIR-SAR evaluates the similarity between a query image and images in the database. The stochastic distance can identify the most similar regions according to the image features, which are the estimated parameters of the data model. Third, the performance of our proposal was evaluated by applying the Mean Average Precision (MAP) measure and considering clippings from three radar sensors, i.e., UAVSAR, OrbiSaR-2, and ALOS PALSAR. The CBIR-SAR results for synthetic images achieved the highest MAP value, retrieving extremely heterogeneous regions. Regarding the real SAR images, CBIR-SAR achieved MAP values above 0.833 for all polarization channels for image samples of forest (UAVSAR) and urban areas (ORBISAR). Our results confirmed that the proposed method is sensitive to the degree of texture, and hence, it relies on good estimates. They are inputs to the stochastic distance for effective image retrieval.

## 1. Introduction

Content-Based Image Retrieval (CBIR) is an important research area in computer vision and information retrieval. It enables users to search for images based on their visual content rather than relying on text-based descriptions or metadata. CBIR systems use features extracted from images to compute the similarity between a reference image and other samples in a database. In the case of SAR images, CBIR can be challenging due to the unique properties of the data, such as the speckle noise, number of looks and spatial resolution to name a few. In the literature, there are different techniques to perform CBIR using SAR images [1,2,3,4,5,6,7].

SAR systems offer advantages as they are not dependent on solar light and atmospheric conditions [8,9]. However, analyzing SAR images is difficult due to the multiplicative granulation caused by speckle, which results from the interference of electromagnetic waves. Although filters can be applied to reduce this effect, it may come at the cost of reduced image quality. Therefore, statistical modeling techniques are used to process and interpret these images. Here, the $G_{I}^{0}$ distribution was adopted to model intensity SAR data [10,11,12] and to develop the CBIR-SAR.

In general, the CBIR systems available in the literature use similarity measures that do not consider that image regions can be discriminated using the estimated parameters of the image model. In fact, it is possible to understand an image as a set of regions that can be described by different probability laws [13]. Thus, it is possible to use stochastic distances to statistically discriminate distributions for SAR image retrieval purposes.

Thus, the aim of this paper is to advance the state-of-the-art on the use of a stochastic distance to overcome the limitation of the classic CBIR to deal with SAR images due to the speckle noise. To achieve this goal, the triangular stochastic distance measures the similarity between regions with different roughness levels or textures based on the parameters of the $G_{I}^{0}$ distribution to develop the CBIR-SAR.

To estimate the parameters of the $G_{I}^{0}$ model, the Maximum Likelihood Estimation (MLE) [14] and the Fast Log-Cumulants Method (FLCM) [12] are applied to the intensity images. Additionally, a stochastic similarity measure uses the estimated parameters [13,15], which are the SAR image features, to distinguish image regions. After obtaining the distances, they are arranged in the ascending order and generate a ranking list of image regions. Then, the performance of the information retrieval system is evaluated by using the Mean Average Precision (MAP) [16].

The main contributions of this work are as follows: (a) development of a reproducible CBIR system based on the intensity SAR data model that evaluates two estimation parameter methods as image feature extractors; and (b) establishment of a knowledge-based information retrieval inspired by stochastic distances and data modeling extensible to other statistical models and stochastic distances.

This paper unfolds as follows: Section 2 describes the fundamental background required to develop the proposed approach. Section 3 introduces a system for content-based SAR image retrieval using a stochastic distance. Section 4 shows the results and discussions, and Section 5 summarizes our findings and conclusions.

## 2. Background on SAR Data Modeling

This section presents the statistical model for intensity SAR images, i.e., the $G_{I}^{0}$ distribution, the parameter estimation methods, and the stochastic distance.

### 2.1. Statistical Model for Intensity SAR Data

**Figure 1:**
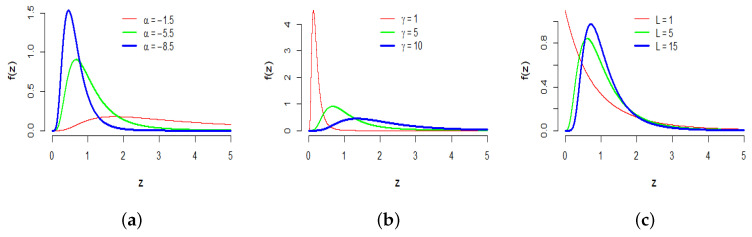
Densities of the $G_{I}^{0}$ distribution with different parameters. (**a**) α∈{−1.5,−5.5,−8.5}, *L* = 8, γ=5, (**b**) γ∈{1,5,10},L=8,α=−5.5 and (**c**) L∈{1,5,15},α=−5.5,γ=5.

Here, the $G_{I}^{0}$ distribution was adopted to model SAR data in intensity. The probability density function of the $G_{I}^{0}$ is defined as [10]:

(1) $$f_{z}\left(z;\alpha ,\gamma ,L\right)=\frac{L^{L}\Gamma \left(L-\alpha \right)}{\gamma^{\alpha }\Gamma \left(-\alpha \right)\Gamma \left(L\right)}z^{L-1}{\left(\gamma +Lz\right)}^{\alpha -L},$$

with −α,γ,L,z>0. The parameters α and γ correspond to the roughness and scale, respectively, Γ(.) is the gamma function defined as [17]:

(2) $$\Gamma (w)=\frac{1}{we^{\tau w}\prod_{k=1}^{\infty }\left(1+\frac{w}{k}\right)e^{-\frac{w}{k}}},$$

with w>0 and the *L* parameter is known as the number of looks, which represents the speckle control parameter. The influence of the parameters (α,γ,L) in the shape of the $G_{I}^{0}$ distribution can be seen in Figure 1.

Figure 1a shows the effect of varying the parameter α for the same L=8 and γ=5. Notice that when the value of α decreases, the probability density function tends to be more homogeneous, representing image regions that are more homogeneous. Figure 1b shows the effect of varying the parameter γ for the same α=−5.5 and L=8. In [18], the γ parameter is related to the brightness of the scene.

Finally, Figure 1c shows the effect of the number of looks, i.e., varying the parameter *L* for the same α=−5.5 and γ=5. It also exhibits that the number of looks has an effect mostly on the distribution of very small values. Notice that when *L* decreases, the $G_{I}^{0}$ distribution assigns more probability to small values of the return *z*, yielding less contrasted images [19] and consequently more challenging images.

The great advantage of using the $G_{I}^{0}$ distribution is that it can accurately model homogeneous, heterogeneous and extremely heterogeneous regions of an intensity SAR image [10]. This flexibility has motivated some important applications, such as SAR image segmentation driven by the $G_{I}^{0}$ Shannon entropy [20] and autoregressive moving average process for speckled data [21], among others.

### 2.2. Parameter Estimation for the $G_{I}^{0}$ Distribution

To estimate the parameters of the $G_{I}^{0}$ distribution [10], two methods were selected: the Maximum Likelihood Estimation [13] and the Fast Log-Cumulants Method [12]. Here, the number of looks, *L*, is considered known. The *L* parameter is generally an integer provided by the SAR sensor, and in this paper, it is a priori information. However, in the absence of it, the number of looks can be estimated from real SAR data, and it is therefore interesting to call the equivalent number of looks [22].

#### 2.2.1. Maximum Likelihood Estimation (MLE)

Consider a random variable *Z* with probability density function f(z,θ), where θ is the vector of parameters to be estimated and ($z_{1},z_{2},\ldots ,z_{n}$) is a random sample of size *n* from the variable *Z*. The likelihood function $L(\theta ;z_{1},z_{2},\ldots ,z_{n})$ is defined by [14]:

(3) $$L\left(\theta ;z_{1},z_{2},\ldots ,z_{n}\right)=\prod_{i=1}^{n}f\left(z_{i},\theta \right),$$

which is interpreted as a function of θ. The maximum likelihood estimator of θ will be the one that maximizes $L(\theta ;z_{1},z_{2},\ldots ,z_{n})$.

For the $G_{I}^{0}$ distribution, the likelihood function is given by [13]:

(4) $$L_{G_{I}^{0}}\left(\alpha ,\gamma ,L;z\right)={\left(\frac{L^{L}\Gamma \left(L-\alpha \right)}{\gamma^{\alpha }\Gamma \left(-\alpha \right)\Gamma \left(L\right)}\right)}^{n}\prod_{i=1}^{n}z_{i}^{L-1}{\left(\gamma +Lz_{i}\right)}^{\alpha -L}.$$

The values of α and γ that maximize Equation (4) can be obtained by finding the solution of the following system of nonlinear equations.

(5) $$\left\{\begin{array}{c} n\left[-\psi^{0}\left(L-\hat{\alpha }\right)-\log\left(\hat{\gamma }\right)+\psi^{0}\left(-\hat{\alpha }\right)\right]+\sum_{i=1}^{n}\log\left(\hat{\gamma }+Lz_{i}\right)=0\\ -\frac{n\hat{\alpha }}{\hat{\gamma }}+\left(\hat{\alpha }-\hat{L}\right)\sum_{i=1}^{n}\frac{1}{\hat{\gamma }+Lz_{i}}=0, \end{array}\right.$$

where $\Psi {\left(.\right)}^{0}$ is the digamma function [17,23] and $\hat{\alpha }$ and $\hat{\gamma }$ are the maximum likelihood estimators for α and γ, respectively. Given that there are no feasible inverse functions for $\psi^{0}\left(.\right)$, the nonlinear system in Equation (5) cannot be solved explicitly, and therefore, it requires iterative procedures. In fact, it is a difficult task, in particular in textureless areas [19].

In order to solve this problem here, we applied a numerical routine based on the Broyden–Fletcher–Goldfarb–Shanno algorithm (BFGS), which was implemented in R software through the *maxLik* function. A good solution consists of using estimates obtained with the Method of Moments [10] as the initial guess for the BFGS algorithm. The algorithm BFGS starts at initial estimates for the optimal values of $\hat{\alpha }$ and $\hat{\gamma }$ and proceeds iteratively to search for better estimates at each stage.

#### 2.2.2. Fast Log-Cumulants Method (FLCM)

This method presents analytical expressions for estimating the roughness (α) and scale (γ) parameters of the $G_{I}^{0}$ distribution. Using FMLC, the roughness parameter can be estimated by the following expression [12]:

(6) $$\hat{\alpha }=\left\{\begin{array}{cc} -\left|\sqrt{\frac{1}{{\tilde{k}}_{2}-\Psi^{1}\left(L\right)}}\right|,if\; & {\tilde{k}}_{2}-\Psi^{1}\left(L\right)<0\\ -\sqrt{\frac{1}{{\tilde{k}}_{2}-\Psi^{1}\left(L\right)}},if\; & {\tilde{k}}_{2}-\Psi^{1}\left(L\right)>0, \end{array}\right.$$

where |.| stands for the modulus operator, $\Psi {\left(.\right)}^{1}$ is the trigamma function [17] and ${\tilde{k}}_{2}={\tilde{m}}_{2}-{\tilde{m}}_{1}^{2}$ is the log-cumulant of order 2. Usually, $\left({\tilde{m}}_{1},{\tilde{m}}_{2}\right)$ must be replaced by the corresponding sample log-moment of order v=1 and v=2, respectively, which can be calculated as [24]:

(7) $${\hat{\tilde{m}}}_{v}=\frac{1}{n}\sum_{i=1}^{n}\ln z_{i}^{v},$$

with $z_{i}$, i∈{1,2,…,n} being a sample of a random variable *Z*. After obtaining $\hat{\alpha }$, the γ parameter can be estimated by:

(8) $$\hat{\gamma }=\exp\left[{\tilde{k}}_{1}-\Psi^{0}\left(L\right)+\Psi^{0}\left(-\hat{\alpha }\right)\right]L,$$

where ${\tilde{k}}_{1}={\tilde{m}}_{1}$ and $\Psi^{0}\left(.\right)$ is the digamma function [17].

### 2.3. Stochastic Distance

Contrast analysis often addresses the problem of quantifying how distinguishable two image regions are from each other [13], and a distance metric plays a crucial role to perform it. Similarly, CBIR systems rely on image content similarity and require distance metrics. Due to the statistical properties of SAR data, CBIR systems require suitable distances. In fact, stochastic distances are relevant tools for SAR image analysis and understanding, since they are capable of assessing differences between regions in a scene.

In this paper, the triangular distance was selected due to its favorable properties, such as not requiring an extreme computational effort and being effective for both extremely heterogeneous and homogeneous regions in SAR images [25]. Similarly to [13], we deal with distances between the same distributions, and thus, only their parameters are relevant. The triangular distance is described as:

(9) $$\begin{array}{c} d_{T}\left(Z_{1},Z_{2}\right)=\int \left(\frac{{\left(f_{Z_{1}}\left(z,\theta_{1}\right)-f_{Z_{2}}\left(z,\theta_{2}\right)\right)}^{2}}{f_{Z_{1}}\left(z,\theta_{1}\right)+f_{Z_{2}}\left(z,\theta_{2}\right)}\right)dz, \end{array}$$

where $Z_{1}$ and $Z_{2}$ are random variables defined in the same probability space, with probability density functions $f_{Z_{1}}\left(z,\theta_{1}\right)$ and $f_{Z_{2}}\left(z,\theta_{2}\right)$, respectively, where $\theta_{1}$ and $\theta_{2}$ are arrays of parameters.

## 3. Proposed Methodology

The proposed CBIR method is based on the knowledge that comes from the statistical model of real SAR images. It uses intensity images acquired from synthetic aperture radar. The database consists of 25 clipping of images of each region (e.g., water, oil spill, forest) from UAVSAR and 25 images of each region (e.g., water, urban area, and forest) from OrbiSAR-2 and ALOS PALSAR. The Maximum Likelihood Estimation and the fast approach of the Log-Cumulative Method are the methods that estimate the roughness and scale parameters for each image region. The number of looks provided by the SAR sensors is equal to 1. Table 1 presents this radar information. The reason for dealing with single-look images is that they are markedly affected by speckle and pose potential challenges for the algorithm. Thus, all tests are performed with single-look images.

**Figure 2:**
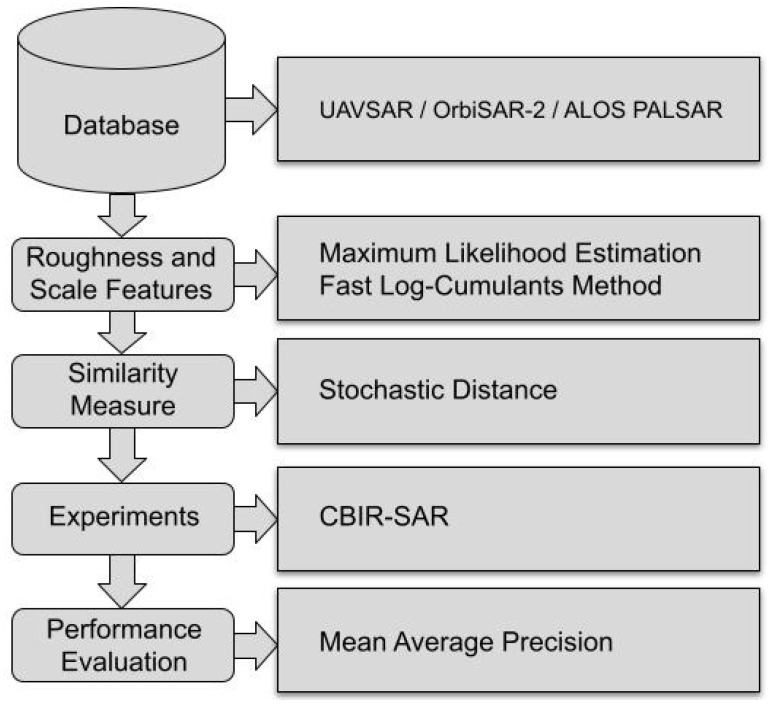
Flowchart of the proposed methodology.

Figure 2 depicts the overall pipeline of the proposed CBIR. It shows that for the SAR image database, the parameters of the $G_{I}^{0}$ distribution for each image region are estimated using MLE and FLCM. The main reason for using these parameter estimation methods is to assess the performance of the proposed algorithm on each one.

The estimates and the similarity measure are inputs to CBIR-SAR to compute the triangular stochastic distance between each query image and the other samples. Finally, the system outputs distances which provide a ranking list of the retrieved samples of water, oil spill, forest and urban area. Then, the MAP measure assesses the results. The higher the MAP value is, the better the performance of CBIR-SAR.

### 3.1. SAR Database

The real SAR images were acquired from three different radars: single-look images from the airborne Uninhabited Aerial Vehicle Synthetic Aperture Radar (UAVSAR) from the National Aeronautics and Space Administration (NASA); OrbiSAR-2 from BRADAR; and ALOS Phased Array type L-band Synthetic Aperture Radar (ALOS PALSAR) from the National Aeronautics and Space Administration (NASA). To create the database, each image was manually cut and categorized into three regions according to the coverage area. The set of samples comprises 25 patches of 100 × 100 pixels for each region of each radar polarization channel, i.e., HH, HV, VH and VV. The VH channel of ALOS PALSAR was unavailable.

**Figure 3:**
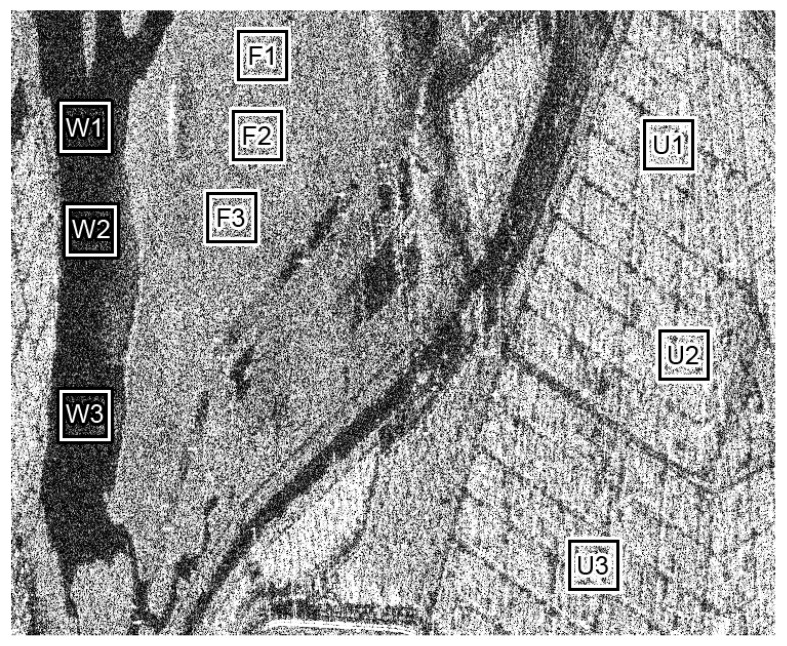
OrbiSAR-2 SAR image: Sample patches selected from water (W), forest (F) and urban areas (U).

Figure 3 exhibits samples of patches, where W1,W2,W3 correspond to water, F1,F2,F3 are forest regions and U1,U2,U3 are urban areas. Table 1 presents important physical information about the three radars that acquired the real SAR images.

### 3.2. Parameter Estimation

In this paper, the MLE and FLCM methods estimate the roughness and scale parameters of the $G_{I}^{0}$ model for each image sample. In terms of CBIR-SAR, the parameter estimation extracts statistical information of images, and it corresponds to the feature extraction step of a CBIR system. Finally, the parameter estimates are inputs to the triangular stochastic distance. Given an image as an input query, CBIR-SAR calculates the distance between it and all images from the database.

### 3.3. CBIR-SAR

CBIR systems search for similar images in a database by assessing the similarity between the reference image and the other images in the database [26]. It uses visual image features to compute distances and arrange them in ascending order and generate a ranking list of images. Here, the visual features are the roughness ($\hat{\alpha }$) and scale ($\hat{\gamma }$) parameters of the $G_{I}^{0}$ model.

The proposed CBIR based on a stochastic approach recognizes and retrieves SAR image regions such as water, forest, urban areas, and oil spills, taking into account the $G_{I}^{0}$ model and the stochastic distance between same distributions, which is possibly indexed by different parameters. Then, CBIR-SAR performs image retrieval matching samples by statistical similarity.

### 3.4. Mean Average Precision (MAP)

For performance evaluation, the Mean Average Precision (MAP) [16] is used, as:

(10) $$AP\left(Q\right)=\frac{\sum_{i=1}^{N_{R}}\left(P\left(i\right)*F\left(i\right)\right)}{S},$$

where P(i) is the precision until the position *i* of the ranking, and F(i) is equal to 1 if the image *i* of the ranking *i* belongs to the same class as the query image *Q* and 0 otherwise. $N_{R}$ is the number of images in the ranking, and *S* is the number of images of the same class obtained by the query image. The MAP is obtained by the average AP for all images of the ranking within the range (0,1), and higher values indicate better performance.

## 4. Results and Discussion

This section presents the results and discussions based on the experiments carried out with CBIR-SAR applied to synthetic and real SAR images acquired from three different SAR sensors. The statistical computational environment used to carry out the experiments was the R language [27], version 4.1.2, on a machine with a CPU of up to 4.20 GHz and 16 GB of RAM.

### 4.1. Experiments with Synthetic SAR Images

Inspired by [22], the intensity SAR images were simulated using:

(11) $$S=-\frac{\gamma }{\alpha }\Upsilon_{2L,-2\alpha }^{-1}\left(U\right),$$

where *S* represents the simulated image, $\Upsilon_{2L,-2\alpha }^{-1}$ is the inverse function of the *F*-Snedecor distribution, with 2L and −2α being the degrees of freedom and *U* is a random variable with uniform distribution over the interval (0,1). To generate SAR images from Equation (11), the following steps are required:

(i) Define the parameters (α,γ,L).
(ii) Generate *U*, with desirable size, from the uniform distribution over the interval (0,1).
(iii) Put *U* in Equation (11). The result will be a synthetic intensity SAR image, with parameters defined in step (i).

Three different sets of intensity SAR images $\left(S_{1},S_{2},S_{3}\right)$ were simulated using Equation (11), where each set comprises 25 image samples of 100 × 100 pixels. Our simulations considered the α parameter values referring to extremely heterogeneous regions (urban areas) in the interval α∈(−0.35,−1.52) for $S_{1}$ as well as to heterogeneous regions (forest areas). The values are in the interval α∈(−10.14,−23.75) for $S_{2}$ and homogeneous regions (water, oil) are in the interval α∈(−55.79,−71.08) for $S_{3}$. With regard to the other parameters, the γ values were adopted in the interval γ ∈ (1, 7) and *L* = 1. After generating the synthetic SAR image database, it we applied the CBIR methodology described in Section 3. Table 2 presents the results of the experiments. It shows the MAP values using both estimators and three regions with different roughness levels.

The results show the relevance of the texture degree and the parameter estimation to obtain MAP values close to 1.0. The higher the degree of texture, the better the performance of CBIR-SAR. FLCM obtained better results than MLE because the latter encountered a numerical convergence problem in the process. However, MAP reached values above 0.81 for all scenarios.

### 4.2. Experiments with Real SAR Images

In this study, the proposed approach was evaluated using three real images from the SAR database, and 25 clippings of the respective regions of size 100 × 100 were created from each original image, resulting in a total of 75 images for each database. The roughness and scale parameters were estimated using FLCM and MLE. Then, we calculatedthe ranking list based on the obtained stochastic distances and the MAP measure value for each image region.

Table 3 presents the results for the different sensors, polarization channels, and bands. The L-band UAVSAR radar database showed promising results using both estimators and all polarization channels. The method efficiently identified homogeneous regions with consistent results, especially for the VV polarization. This polarization provides better contrast between the oil slick and the ocean, which indicates that it is a suitable channel to detect roughness and brightness variations from the sea surface, such as those caused by oil spills.

The experiments with OrbiSAR-2 achieved the highest MAP values for urban areas for all channels. On the other hand, MAP values around 0.5 indicate that CBIR-SAR incorrectly considered water and forest samples with similar texture. Both MLE and FCLM provided good estimates and hence MAP values above 0.88 for water samples in VV. The HH channel achieved the best result for forest.

The results with ALOS PALSAR for HH and HV were superior to VV, mostly in extremely heterogeneous regions. On the other hand, VV presented the best results for water. Overall, CBIR-SAR performed better in homogeneous regions for both L-band sensors. The worst results for VV refer to heterogeneous regions, i.e., forest.

## 5. Conclusions

The proposed method is based on the statistical data modeling and stochastic distance to perform CBIR on single-look SAR images. Single-look SAR images are more challenging due to the marked presence of the speckle noise. The $G_{I}^{0}$ distribution models the SAR intensity data, and its parameters are estimated by two estimation methods, i.e., the Maximum Likelihood Estimation and the Fast Log-Cumulant Method. The input features of the CBIR system comprise the estimated parameters of the image region, and the triangular distance compares the similarity between image regions. These estimated parameters represent the roughness and mean brightness features which discriminate image regions. In fact, the stochastic distance is highly dependent on the estimation quality of the parameters. Both estimation methods were tested: MLE due to its good properties and FLCM due to its estimation speed.

Our experiments demonstrated the effectiveness of the proposed method with synthetic and real SAR images. For both estimators, CBIR-SAR achieved the highest MAP values for extremely heterogeneous regions. These results confirmed that the proposed method is able to retrieve regions of similar texture based on the roughness parameter and hence, it is highly dependent on the region texture. However, it accomplished satisfactory results for homogeneous regions, and it was capable of revealing subtle texture differences between water and oil spills. Both synthetic and real SAR data results support that the proposed CBIR-SAR provides an effective solution to overcome the challenges posed by speckle for SAR image retrieval. We also demonstrated the role of the statistical data model, estimation methods and stochastic distance in the development of an expert system that can organize and query SAR image regions more efficiently. Tests with other stochastic distances and data may extend the method for other scenarios and applications, such as change detection and classification.

Among the advantages of the proposed methodology, we can list the following. CBIR-SAR is able to retrieve different image regions based on the parameters of the $G_{I}^{0}$ model, which are image features embodied in the stochastic triangular distance. In addition, our approach deals with raw data and hence it does not require a preprocessing step due to the speckle noise. Lastly, the methodology presented promising results on both parameter estimation methods. Moreover, CBIR-SAR results may also drive other image processing techniques, such as SAR image segmentation and classification. A disadvantage of this methodology is that the MLE estimation method requires the use of numerical techniques.

## Figures and Tables

**Table 1:** Radar Information.

**UAVSAR**	Covered Place	New Orleans, LA, USA
Physical Information	Height (km)	265.26
	Width (km)	16.49
	Area (km${}^{2}$)	4374.14
Radar Parameters	Sensor	SAR
	Polarization	HH, HV, VH, VV
	Band	L Band
Data Information	Product Format	SLC
	Number of Rows	442,106
	Number of Columns	9900
	Number of Looks	1
**ORBISAR-2**	Covered Place	Santos, Brazil
Physical Information	Height (km)	265.26
	Width (km)	16.49
	Area (km${}^{2}$)	4374.14
Radar Parameters	Sensor	SAR
	Polarization	HH, HV, VH, VV
	Band	P Band
Data Information	Product Format	SLC
	Number of Rows	26,616
	Number of Columns	8192
	Number of Looks	1
**ALOS PALSAR**	Covered Place	San Francisco, CA, USA
Physical Information	Height (km)	65.92
	Width (km)	28.57
	Area (km${}^{2}$)	1883.33
Radar Parameters	Sensor	SAR
	Polarization	HH, HV, VV
	Band	L Band
Image Data Information	Product Format	CEOS
	Number of Rows	18,432
	Number of Columns	1248
	Number of Looks	1

**Table 2:** MAP results for synthetic images and both estimators.

Region	MLE	FLCM
Extremely Heterogeneous	0.817	0.997
Heterogeneous	0.845	0.847
Homogeneous	0.820	0.859

**Table 3:** Mean Average Precision for all images using MLE and FLCM.

		HH	HV	VH	VV
UAVSAR					
Water	MLE	0.751	0.620	0.713	0.880
	FLCM	0.949	0.959	0.947	0.904
Oil Spill	MLE	0.854	0.302	0.956	0.934
	FLCM	0.943	0.978	0.938	0.990
Forest	MLE	0.995	0.988	1.000	0.833
	FLCM	1.000	0.994	1.000	0.856
ORBISAR					
Water	MLE	0.791	0.694	0.593	0.860
	FLCM	0.926	0.686	0.930	0.864
Urban Area	MLE	1.000	0.984	0.919	0.959
	FLCM	1.000	0.985	0.919	0.957
Forest	MLE	0.845	0.429	0.583	0.573
	FLCM	0.863	0.545	0.627	0.615
ALOS PALSAR					
Water	MLE	0.940	0.987	-	0.833
	FLCM	0.781	0.897	-	0.820
Urban Area	MLE	0.889	0.931	-	0.618
	FLCM	0.877	0.953	-	0.617
Forest	MLE	0.904	0.980	-	0.555
	FLCM	0.821	0.972	-	0.560

- data is not available for this channel.

## Data Availability

No new data were created or analyzed in this study. Data sharing is not applicable to this article.

## References

[1] Krishna G.S., Prakash N. (2021). Deep learning for efficient and multi-labelled classification of synthetic aperture radar images. Evol. Syst..

[2] Alshehri M. (2020). A content-based image retrieval method using neural network-based prediction technique. Arab. J. Sci. Eng..

[3] Zhang K., Li B., Tao R. SAR image retrieval based-on fly algorithm. Proceedings of the Tenth International Conference on Advanced Computational Intelligence (ICACI).

[4] Tang X., Jiao L., Emery W.J. (2017). SAR Image Content Retrieval Based on Fuzzy Similarity and Relevance Feedback. IEEE J. Sel. Top. Appl. Earth Obs. Remote Sens..

[5] Jiao L., Tang X., Hou B., Wang S. (2015). SAR Images Retrieval Based on Semantic Classification and Region-Based Similarity Measure for Earth Observation. IEEE J. Sel. Top. Appl. Earth Obs. Remote Sens..

[6] Tang X., Jiao L. (2017). Fusion Similarity-Based Reranking for SAR Image Retrieval. IEEE Geosci. Remote Sens. Lett..

[7] Schroder M., Rehrauer H., Seidel K., Datcu M. (2000). Interactive learning and probabilistic retrieval in remote sensing image archives. IEEE Trans. Geosci. Remote Sens..

[8] Argenti F., Lapini A., Bianchi T., Alparone L. (2013). A tutorial on speckle reduction in synthetic aperture radar images. IEEE Geosci. Remote Sens. Mag..

[9] Nobre R.H., Rodrigues F.A.A., Marques R.C.P., Nobre J.S., Neto J.F.S.R., Medeiros F.N.S. (2016). SAR Image Segmentation with Renyi’s Entropy. IEEE Signal Process. Lett..

[10] Frery A.C., Muller H.J., Yanasse C.C.F., Sant’Anna S.J.S. (1997). A model for extremely heterogeneous clutter. IEEE Trans. Geosci. Remote Sens..

[11] Gambini J., Cassetti J., Lucini M.M., Frery A.C. (2015). Parameter Estimation in SAR Imagery Using Stochastic Distances and Asymmetric Kernels. IEEE J. Sel. Top. Appl. Earth Obs. Remote Sens..

[12] Rodrigues F.A.A., Nobre J.S., Vigélis R., Liesenberg V., Marques R.C.P., Medeiros F.N.S. A Fast Approach for the Log-Cumulants Method Applied to Intensity SAR Image Processing. Proceedings of the 2020 IEEE Latin American GRSS and ISPRS Remote Sensing Conference (LAGIRS).

[13] Nascimento A.D.C., Cintra R.J., Frery A.C. (2010). Hypothesis testing in speckled data with stochastic distances. IEEE Trans. Geosci. Remote Sens..

[14] Casella G., Berger R.L. (2021). Statistical Inference.

[15] Braga I.H.T., Sacramento V.P., Oliveira L.C.C., Medeiros F.N.S., Rodrigues F.A.Á. (2022). Ocean surface change detection from remote sensing image based on stochastic similarity measure. Braz. J. Water Resour..

[16] Wang B., Brown D., Gao Y., La Salle J. (2015). MARCH: Multiscale-arch-height description for mobile retrieval of leaf images. Inf. Sci..

[17] Arfken G.B., Weber H.J. (1999). Mathematical Methods for Physicists.

[18] Bustos O.H., Lucini M.M., Frery A.C. (2002). M-Estimators of Roughness and Scale for $G_{A}^{0}$-Modelled SAR Imagery. EURASIP J. Appl. Signal Process..

[19] Frery A.C., Wu J., Gomez L. (2022). Sar Image Analysis—A Computational Statistics Approach.

[20] Ferreira J.A., Nascimento A.D.C. (2020). Shannon Entropy for the $G_{I}^{0}$ Model: A New Segmentation Approach. IEEE J. Sel. Top. Appl. Earth Obs. Remote Sens..

[21] Junior P.M.A., Nascimento A.D.C. (2021). $G_{I}^{0}$ ARMA process for speckled data. J. Stat. Comput. Simul..

[22] Marques R.C.P., Medeiros F.N., Nobre J.S. (2012). SAR image segmentation based on level set approach and $G_{A}^{0}$ model. IEEE Trans. Pattern Anal. Mach. Intell..

[23] Abramowitz M., Stegun I.A. (1964). Handbook of Mathematical Functions with Formulas, Graphs, and Mathematical Tables.

[24] Nicolas J.M. (2002). Introduction aux statistiques de deuxième espèce: Applications des logs-moments et des logs-cumulants à l’analyse des lois d’images radar. TS. Trait. Signal.

[25] Rey A., Revollo Sarmiento N., Frery A.C., Delrieux C. (2022). Automatic Delineation of Water Bodies in SAR Images with a Novel Stochastic Distance Approach. Remote Sens..

[26] Liu Y., Zhang D., Lu G., Ma W.Y. (2007). A survey of content-based image retrieval with high-level semantics. Pattern Recognit..

[27] R Core Team (2014). R: A language and environment for statistical computing. MSOR Connect..

